# A History of Childhood Maltreatment Has Substance- and Sex-Specific Effects on Craving During Treatment for Substance Use Disorders

**DOI:** 10.3389/fpsyt.2022.866019

**Published:** 2022-04-14

**Authors:** Sarah Gerhardt, Katharina Eidenmueller, Sabine Hoffmann, Nina K. Bekier, Patrick Bach, Derik Hermann, Anne Koopmann, Wolfgang H. Sommer, Falk Kiefer, Sabine Vollstädt-Klein

**Affiliations:** ^1^Department of Addictive Behavior and Addiction Medicine, Central Institute of Mental Health, Medical Faculty Mannheim, Heidelberg University, Mannheim, Germany; ^2^Department of Biostatistics, Central Institute of Mental Health, Medical Faculty Mannheim, Heidelberg University, Mannheim, Germany; ^3^Therapieverbund Ludwigsmühle gGmbH, Landau, Germany; ^4^Institute for Psychopharmacology, Central Institute of Mental Health, Medical Faculty Mannheim, Heidelberg University, Mannheim, Germany; ^5^Bethania Hospital for Psychiatry, Psychosomatics, and Psychotherapy, Greifswald, Germany; ^6^Mannheim Center of Translational Neurosciences, Medical Faculty Mannheim, Heidelberg University, Mannheim, Germany; ^7^Feuerlein Center on Translational Addiction Medicine, Heidelberg University, Heidelberg, Germany

**Keywords:** childhood trauma, addiction, sex differences, substance craving, substance use disorder, perceived stress, addiction treatment

## Abstract

**Rationale:**

Childhood maltreatment (CM) leads to detrimental mental health outcomes, such as substance use disorders (SUD). This study examined prevalence and severity of all five types of CM with respect to specific substances and sex in treatment-seeking individuals with SUD. The influences of type of CM and symptoms of depressiveness, anxiety, and perceived stress on substance craving at admission as well as craving reduction during SUD treatment were examined.

**Methods:**

*N* = 546 patients in treatment for SUD and *N* = 109 individuals in opioid maintenance treatment filled out questionnaires regarding CM (Childhood Trauma Questionnaire) and psychopathologies. Substance craving was assessed throughout treatment using the Mannheim Craving Scale. Group differences in CM, type of substance and sex were examined. General linear models were applied to examine influences on substance craving.

**Results:**

Higher prevalence and severity of all five subtypes of CM were observed in individuals with SUD compared to the general population. Women were more severely affected by emotional and sexual abuse than men. Patients with cannabis use disorder reported more severe experiences of emotional abuse compared to all other substances. Craving at admission to treatment was influenced by emotional abuse, however, symptoms of depressiveness, anxiety, and perceived stress contributed to craving at admission or craving reduction during treatment.

**Conclusion:**

CM relates to SUD and should be incorporated in prevention and treatment of SUD. Underlying mechanisms of the association might relate to impairments in processing and regulation of stress, emotions, and interpersonal relations following a history of CM.

## Introduction

A variety of studies examined the consequences of adverse childhood experiences (ACE) that are related to the development of somatic and mental disorders (1). ACE are defined as household dysfunction but also childhood maltreatment (CM) (2, 3). Specifically, CM is operationalized as emotional, physical, and sexual abuse as well as emotional and physical neglect (4). A history of CM is related to the age of onset and severity of subsequent mental disorders, and reduces treatment response (4–9).

In Europe, high prevalence rates of CM have been reported for the general population: 29.1% for emotional abuse, 22.9% for physical abuse, 13.4% (female) and 5.7% (male) for sexual abuse, 16.3% for physical neglect and 18.4% for emotional neglect (10). Figures for Germany are comparable, between 6.5% for at least moderate emotional abuse and 22.4% for at least moderate physical neglect (11).

A history of CM is frequently observed in individuals with substance use disorders (SUD) (12–16). It increases the risk of developing a SUD (13, 17–19), and this extends also to non-substance use disorders such as problematic and pathological gambling (20, 21). Compared to the general population in Germany (22), individuals with SUD have experienced more severe forms of CM (23). For example, the prevalence in individuals with opioid use disorder (OUD) ranges between 16% for sexual abuse in men and 43% for emotional abuse (15).

Since prevalence number of SUD and relapse rates after SUD treatment are high [e.g., (24–26)], examining factors contributing to the development and maintenance of SUD are still of importance. A stable, mostly correlational, relation has been observed between CM and different kinds of SUD even after correction for comorbid psychiatric disorders and sociodemographic variables (27). The age of drinking onset was 1 year earlier in individuals with CM (28). Furthermore, exposure to several CM predicted SUD in young adults, irrespectively of sociodemographic variables (e.g., sex or ethnicity) and after controlling for prior mental disorders (29). Similarly, a cumulative effect of the number of types of CM events was observed regarding the severity of alcohol use disorder (AUD) (30). Regarding all five sub-types of CM, emotional abuse is the strongest predictor for the severity of AUD, followed by physical abuse (31). Further, women with CM, compared to women without CM or men, were observed to have a shorter timespan between onset of drinking and AUD and lower rates of abstinence after AUD treatment were associated with CM (28, 32). Contributing to this relation, it has been observed, that the association between cumulative CM and SUD was partly mediated by mood- and anxiety disorders that preceded SUD (33).

Besides CM being associated with SUD, substance craving contributes to relapse (34–37) and, thus, maintenance of the disorder. Further, an effect of stress on substance craving was observed for methadone (38), cocaine (39), or alcohol (40), possibly linking CM, if seen as early life stress, to craving and relapse (41).

Despite the above-mentioned impact of CM on characteristics of SUD, to our knowledge no study examined CM in individuals seeking treatment for SUD while directly comparing different SUDs, investigating sex effects, or addressing the influence of the type of CM on substance craving.

Within the current project we hypothesized that (1) in individuals with SUD, prevalence of all forms of CM is higher in individuals with OUD compared to all other substances; that (2) the severity of CM is strongest in individuals with OUD compared to all other substances. For both (1, 2) women are more severely affected than men. We further hypothesize that (3) in SUD, the severity of CM is positively associated to the severity of depressive and anxious symptoms, and perceived stress; that (4) emotional abuse followed by physical abuse are predictors for the severity of craving at admission to SUD treatment; and that (5) experiences of emotional abuse and physical abuse hamper the decrease of substance craving during SUD treatment while sex and type of SUD but not age exert an effect on the latter two relationships (hypotheses 4 and 5).

## Materials and Methods

### Procedure and Participants

The aggregated dataset (*N* = 655 individuals) derives from two sources. Firstly, between 2016 and 2020, individuals with different kinds of SUD (*N* = 546, sample 1) participated in a questionnaire-based examination during their treatment in the Clinic of Addictive Behavior and Addiction Medicine, Central Institute of Mental Health, Mannheim, Germany. In either an inpatient or a day care setting they received a detoxification and a psychological SUD-related treatment including motivational and cognitive behavioral elements with the goal of continuous abstinence (42). SUD patients filled out several questionnaires at admission and once weekly during the treatment period of 24 ± 9.7 days. In case of repeated admissions during the data collection period of 2016 and 2020, the most recent admission time point was chosen. Diagnoses of substance addiction and additional comorbid mental disorders were made by trained medical staff following the International Classification of Diseases (ICD-10). Regarding SUD as described in the Diagnostic and statistical manual of mental disorders, 5th version (DSM-5) (43), substance addiction corresponds to moderate to severe SUD (44).

Secondly, data (*N* = 109, sample 2) from a research project including outpatients of the opioid maintenance treatment (OMT) of the Central Institute of Mental Health, Mannheim, were included to enrich the first dataset with individuals suffering from OUD. Data collection and diagnostic procedures also were performed by trained medical staff and a senior psychiatrist. A study description of sample 2 has previously been published (45).

For all individuals (samples 1 and 2), general inclusion criteria were: age over 18 years, sufficient knowledge of the German language (oral and in writing), main diagnosis of SUD and availability of data regarding the CM. Please see Supplementary Figure 1 for details of the data collection, preparation and allocation process.

The local Ethics Committee of the Medical Faculty Mannheim, Heidelberg University, Germany, approved the here presented study procedures (approval number 2018-531N-MA and 2018-807R-MA). Information for the first dataset (sample 1) was collected during the patients’ inpatient treatment for clinical purpose and later used for retrospective analyses. Following the recommendation of the ethics committee to protect data privacy the data set was anonymized. Regarding the second dataset (sample 2), in accordance with the Declaration of Helsinki, all participants provided written informed consent prior to study participation.

### Measures

As the focus of this study, all five sub-types of CM, namely emotional, physical, and sexual abuse as well as emotional and physical neglect, were assessed retrospectively using the reliable (0.87 < alpha < 0.95) childhood trauma questionnaire (CTQ), a previously validated self-report questionnaire that addresses the childhood up to the age of 18 years (46). All items of the German version were answered on a 5-point Likert scale (“not at all” to “very often”) leading to sum scores between 5 (no CM) and 25 (severe form of CM) for each subscale, respectively (23). As reported by others (11, 47, 48), the severity of each subscale of CM was additionally described by aggregating the CTQ score for each subscale separately into none-minimal, minimal-moderate, moderate-severe and severe-extreme. Further, prevalence was calculated following Witt et al. (11). To do so, all subscales of the CTQ were dichotomized into “having experienced this form of CM” including moderate to extreme CM and “not having experienced this form of CM” including none to moderate CM. The number of overall CM was calculated by summing up affirmed, dichotomized CTQ subscales.

To characterize sample 1 (*N* = 546), besides assessing the main diagnosis of SUD and sociodemographic variables (e.g., age, gender, employment, marital status, and education), additional questionnaires were administered. The CTQ, Perceived Stress Scale (PSS) (49), and Fagerstrom Test for Nicotine Dependence (FTND) (50) were administered only once, at least 1 week after admission. The Beck Depression Inventory (BDI) (51, 52), Beck Anxiety Inventory (BAI) (53), and Mannheimer Craving Scale (MACS) (54) were administered at admission and every 7 days during treatment. The MACS retrospectively measures overall craving during the last 7 days independent of the substance and has shown to be highly reliable (0.87 < alpha < 0.93). MACS was applied at admission, after 1 and 2 weeks (at T01, T07, and T14), respectively. The reduction of craving after 2 weeks as the difference T01 minus T14 was used to address the course of the treatment. Regarding sample 2 of *N* = 109 OMT individuals, the same sociodemographic variables were assessed and the CTQ was administered.

### Analyses and Statistics

The main SUD diagnosis was grouped into six categories: alcohol use disorder (AUD), cannabis use disorder (CUD), cocaine and stimulant use disorder (CSUD), sedative, hypnotics, or anxiolytic use disorders (SHA), opioid use disorder (OUD, sample 1 only), and opioid use disorder during opioid maintenance (OMT; sample 2 only). OMT and OUD samples were compared using independent samples *t*-tests and chi-square tests including available data for both samples to justify merging both data sets (samples 1 and 2, OUD + OMT) analyses including the CTQ (see Supplementary Material).

A sample description was created, and group differences were examined using analyses of variance (ANOVA) or Welch-Test for continuous data, and chi-square tests for dichotomous data. *Post hoc* tests included Tukey’s or Games–Howell tests for ANOVAs and Welch-Tests. Adjusted *z*-scores and a transformation into *p*-values were performed using chi-square tests according to García-pérez and Núñez-antón (55). Further, the total number of additional SUD diagnoses and a dichotomous item on comorbid mental disorders (yes/no) were calculated. Relevant clinical variables (i.e., CM, substance craving, and symptoms of depressiveness or anxiety, perceived stress) were correlated pairwise (Pearson correlation) to assess bi-directional relations within the overall sample and separated by sex. General linear models (GLM, univariate) were used to assess the influences of CM and clinical variables (i.e., symptoms of depressiveness or anxiety, perceived stress) as well as sociodemographic variables (i.e., age and sex) on the SUD outcome (i.e., substance craving at admission, reduction of craving over the first 2 weeks of treatment). Descriptive and statistical analyses were performed in SPSS (Statistics for Windows, Version 27.0, IBM Corp., Armonk, NY, United States). To counteract multiple testing problems and following Storey (56) false discovery rate (FDR) using the Benjamini and Hochberg method was applied when adequate and results were reported when surviving the correction (*p* < 0.05).

## Results

### Sample Composition

Out of *N* = 1,599 data sets, *N* = 804 data sets with information regarding the CTQ questionnaire (50%) were available. After excluding duplicate data sets due to readmission (*N* = 78) and individuals without a main diagnosis of SUD (*N* = 72), *N* = 655 data sets were available for subsequent analyses (41%), see flow-chart in the Supplementary Material. Between January 2016 and December 2020, *N* = 655 individuals provided information regarding the CTQ and additional questionnaires. Data were collected from the day care clinic (*N* = 391), the inpatient treatment (*N* = 136) and the outpatient opioid maintenance program (*N* = 109).

Participants were between 18 and 86 years of age (mean = 42.0 ± 13.0). They were mostly male (73.3%), single (51.0%) and had no children (40.9%). They received primary and secondary education of 12.8 years, but more than half were currently not steadily employed (57.4%). The majority of participants were tobacco smokers (74.8%). In sample 1, 66.7% (*N* = 364) were diagnosed with AUD as the main diagnosis, 21.6% (*N* = 118) with CUD, 7.8% (*N* = 43) with CSUD, 2.2% (*N* = 12) with SHA, and 1.6% (*N* = 9) with OUD, respectively. Sociodemographic and clinical variables differed between substance groups. See Tables 1, 2 for more details regarding sociodemographic and clinical information.

**TABLE 1:** Sociodemographic data of the overall sample.

	AUD	CUD	CSUD	SHA	OUD + OMT	Descriptive statistics
*N*	364 (55.6%)	118 (18.0%)	43 (6.6%)	12 (1.8%)	118 (18.0%)	655
Age	**47.23 (12.66)^1,2,3^**	**28.6 (7.5)^1,4,5,6^**	**33.3 (7.2)^2,4,7,8^**	**42.9 (9.7)^5,7^**	**42.1 (8.1)^3,6,8^**	***F*(4,68.6) = 103.59, *P* < 0.00**
Gender (male, %)	74.2	73.7	74.4	58.3	71.2	χ^2^(4) = 0.90, *p* = 0.824
Family status (single yes, %)	37.6	78.0	69.8	50.0	65.7	**χ ^2^(4) = 69.30, *p* < 0.001^a^**
Children (yes, %)	42.0	22.9	32.6	28.6	46.5	χ^2^(4) = 16.38, *p* < 0.001^a^
Years of education	**13.5 (2.7)^1,2,3^**	**12.4 (2.6)^1,4^**	**12.2 (2.6)^2^**	13.8 (2.9)	**11.3 (2.4)^3,4^**	***F*(4,561) = 14.90, *p* < 0.001**
Employed (yes, %)	36.8	31.4	23.3	22.2	19.7	**χ ^2^(4) = 22.60, *p* < 0.001*^a^***

*Mean values (standard deviation) or percentage values are displayed. Group differences are highlighted. N, total sample size; AUD, alcohol use disorder; CSUD, cocaine and stimulant use disorders; CUD, cannabis use disorder; SHA, sedative, hypnotics, or anxiolytic use disorders; OUD + OMT, opioid use disorders + opioid maintenance treatment. ^1,2,3,4,5,6,7,8^Superscripted numbers describe significant group differences following post hoc tests. ^a^Following post hoc testing including correction for multiple comparison, no statistically significant group-differences emerged. Significant results are highlighted in bold.*

**TABLE 2:** Clinical data of sample 1.

Sample 1	AUD	CUD	CSUD	SHA	OUD	Descriptive statistics
*N*	364	118	43	12	9	546
Type of stay (inpatient:day care-clinic, %)	26.9:73.1	21.2:78.8	20.9:79.1	58.3:41.7	44.4:55.6	**χ ^2^(4) = 9.70, *p* = 0.021*^a^***
Mental comorbidities, current (yes, %)	47.5	51.7	48.8	66.7	88.9	χ^2^(4) = 7.72, *p* = 0.103
Mental comorbidities, lifetime (yes, %)	56.6	56.8	55.8	75.0	88.9	χ^2^(4) = 5.33, *p* = 0.255
Total number of SUD, current	**1.8 (0.9)^1,2^**	**2.5 (1.0)^1^**	**2.7 (1.2)^2^**	2.8 (1.3)	3.1 (1.4)	***F*(4,33.4) = 14.94, *p* < 0.000**
Total number of SUD, lifetime	**2.0 (1.0)^1,2^**	**2.7 (1.1)^1^**	**3.2 (1.4)^2^**	2.8 (1.5)	3.1 (1.4)	***F*(4,33.5) = 14.87, *p* < 0.001**
Smokers (yes, %)	59.6	79.7	65.1	75.0	93.2	**χ ^2^(4) = 54.62, *p* < 0.001^a^**
FTND of smokers^b^	5.3 (2.4)	4.9 (2.2)	5.3 (2.0)	5.0 (1.8)	5.6 (1.4)	*F*(4,359) = 0.494, *p* = 0.740
BDI at admission	**18.9 (11.8)^1^**	**25.2 (12.0)^1^**	21.1 (11.5)	29.2 (10.2)	25.2 (12.0)	***F*(4,466) = 6.94, *p* < 0.001**
BAI at admission	**16.9 (13.0)^1^**	19.4 (13.0)	**15.9 (10.2)^2^**	**31.5 (11.0)^1,2^**	19.4 (13.0)	***F*(4,459) = 3.49, *p* = 0.008**
PSS^b^	**20.8 (6.3)^1^**	**23.5 (5.4)^1^**	22.5 (5.8)	24.4 (5.4)	23.5 (5.4)	***F*(4,406) = 4.08, *p* = 0.003**
MACS at admission	**16.6 (9.8)^1,2^**	**20.4 (10.2)^1^**	**21.0 (9.4)^2^**	25.0 (8.9)	19.8 (7.7)	***F*(4,467) = 5.27, *p* < 0.001**

*Mean values (standard deviation) or percentage values are displayed for the clinical sample only. Group differences are highlighted. n, sample size; AUD, alcohol use disorder; BAI, Beck Anxiety Inventory; BDI, Beck Depression Inventory; CSUD, cocaine and stimulant use disorders; CUD, cannabis use disorder; FTND, Fagerstrom Test for Nicotine Dependence; MACS, Mannheimer Craving Scale; SHA, sedative, hypnotics, or anxiolytic use disorders; SUD, substance use disorder; OUD, opioid use disorders; PSS, perceived stress scale. ^1,2^Superscripted numbers describe significant group differences following post hoc tests. ^a^Following post hoc testing including correction for multiple comparison, no statistically significant group-differences emerged. ^b^Only administered once. Significant results are highlighted in bold.*

### Prevalence and Severity for All Sub-Types of Childhood Maltreatment With Respect to Different Kinds of Substance Use Disorders

Over all substances, prevalence rates of CM were 19.1% for sexual abuse, 19.8% for physical abuse, 24.7% for emotional abuse, 54.7% for physical neglect, and 67.9% for emotional neglect. Individuals with SUD experienced on average 1.90 (1.46) of five types CM, and significant group differences between substances emerged [*F*(4,540) = 4.48, *p* = 0.001]. *Post hoc* tests indicated a significant difference in the number of CM between AUD [on average 1.71 (1.42) CM] and CUD [on average 2.38 (1.44) CM].

Within the overall sample, severity of CM [mean of sum scores (standard deviation)] resulted in 6.2 (3.5) for sexual abuse, 7.7 (4.4) for physical abuse, 8.8 (3.6) for physical neglect, 10.0 (5.4) for emotional abuse, and 13.2 (5.7) for emotional neglect. Significant group differences with respect to the main diagnosis were observed for emotional abuse [*F*(4,622) = 14.29, *p* < 0.001] and physical abuse [*F*(4,52.5) = 5.09, *p* = 0.001]. *Post hoc* tests indicated significantly more severe experience of emotional abuse for CUD compared to AUD and OUD, and, additionally, of emotional neglect for CUD compared to AUD. See Table 3 for details regarding prevalence for and severity of specific subtypes of CM in different substances.

**TABLE 3:** Severity of childhood maltreatment.

	AUD	CUD	CSUD	SHA	OUD + OMT	Statistics
*N*	364	118	43	12	118	655
CTQ sum score	**43.2 (16.6)^1^**	**51.4 (17.5)^1^**	43.7 (15.2)	42.6 (13.7)	46.9 (17.7)	***F*(4,539) = 4.96, *p* = 0.001**
Number of types of CM	**1.71 (1.42)^1^**	**2.38 (1.44)^1^**	1.70 (1.41)	1.71 (1.98)	1.99 (1.48)	***F*(4,540) = 4.48, *p* = 0.001**
CTQ emotional abuse	**9.2 (5.2)^1^**	**12.8 (6.0)^1,2^**	10.4 (5.2)	9.4 (3.9)	**9.5 (4.7)^2^**	***F*(4,622) = 14.29, *p* < 0.001**
Prevalence (yes, %)	19%	47%	28%	25%	19%	**χ ^2^(4) = 35.18, *p* < 0.001^a^**
CTQ emotional neglect	12.7 (5.6)^1^	14.5 (5.6)^1^	13.0 (6.1)	12.7 (5.7)	13.5 (5.9)	*F*(4,625) = 2.16, *p* = 0.072
Prevalence (yes, %)	65%	78%	62%	50%	70%	χ^2^(4) = 6.48, *p* = 0.166
CTQ physical abuse	7.3 (4.0)	**8.2 (5.6)^1^**	7.9 (4.3)	**6.0 (1.5)^1,2^**	**8.7 (5.2)^2^**	***F*(4,52.5) = 5.09, *p* = 0.001**
Prevalence (yes, %)	16%	24%	26%	8%	27%	**χ ^2^(4) = 10.58, *p* = 0.032^a^**
CTQ physical neglect	8.7 (3.4)	9.2 (4.0)	8.0 (3.1)	8.6 (2.8)	9.1 (3.8)	*F*(3,627) = 1.37, *p* = 0.241
Prevalence (yes, %)	54%	58%	47%	50%	56%	χ^2^(4) = 2.413, *p* = 0.660
CTQ sexual abuse	6.0 (3.3)	6.6 (4.0)	5.5 (1.5)	6.5 (3.7)	6.7 (4.0)	*F*(4,617) = 1.72, *p* = 0.144
Prevalence (yes, %)	15%	28%	12%	25%	25%	**χ ^2^(4) = 13.093, *p* = 0.011^a^**

*Mean values (standard deviation) or percentage values are displayed. Group differences are highlighted in bold. n, sample size; AUD, alcohol use disorder; CSUD, cocaine and stimulant use disorders; CTQ, Childhood Trauma Questionnaire; CUD, cannabis use disorder; SHA, sedative, hypnotics, or anxiolytic use disorders; OUD, opioids use disorders + opioid maintenance treatment. Prevalence numbers and the number of types of CM are reported for the dichotomized item “having experiences CM” coding “yes” for at least moderate experience of the respective subscale of CM. ^1,2^Superscripted numbers describe significant group differences following post hoc tests.*

*^a^Following post hoc testing including correction for multiple comparison, no statistically significant group-differences emerged.*

### Sex Differences in Prevalence and Severity of Childhood Maltreatment

Over all substances, females in comparison to males reported significantly more often having experienced emotional abuse [χ^2^(1) = 26.31, *p* < 0.001], physical abuse [χ^2^(1) = 9.19, *p* = 0.002] and sexual abuse [χ^2^(1) = 37.71, *p* < 0.001], but not emotional neglect [χ^2^(1) = 0.46, *p* = 0.423] or physical neglect [χ^2^(1) = 1.66, *p* = 0.197]. Depending on the main diagnosis, significant sex differences to the detriment of women became apparent for alcohol and emotional abuse [χ^2^(1) = 14.45, *p* < 0.001], alcohol and physical abuse [χ^2^(1) = 7.09, *p* = 0.008], alcohol and sexual abuse [χ^2^(1) = 12.38, *p* < 0.001], cannabis and emotional abuse [χ^2^(1) = 7.28, *p* = 0.007], cannabis and physical abuse [χ^2^(1) = 5.94, *p* = 0.015], cannabis and sexual abuse [χ^2^(1) = 11.15, *p* = 0.001] and opioids and sexual abuse [χ^2^(1) = 9.09, *p* = 0.003].

**FIGURE 1:**
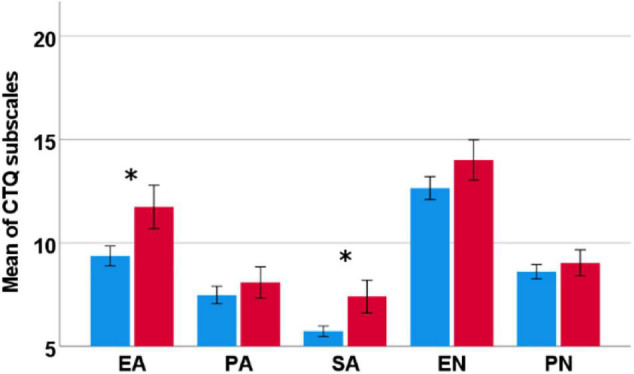
Significant sex differences for the overall sample regarding mean values of the sum scores per subscale of the CTQ. Females (red) reported significantly more severe CM for emotional and sexual abuse than males (blue). CTQ, Childhood Trauma Questionnaire; EA, emotional abuse; PA, physical abuse, SA, sexual abuse, EN, emotional neglect; PN, physical neglect. Error bars are displayed at a 95% confidence interval. *Significant sex difference.

**FIGURE 2:**
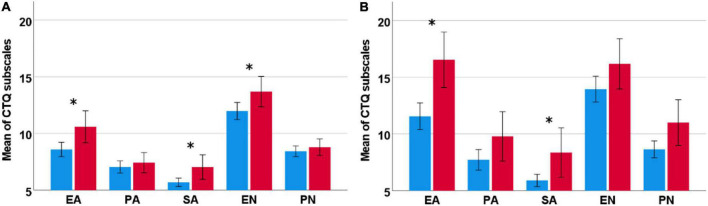
Significant sex differences for the main diagnoses AUD (left) and CUD (right) regarding mean values of the sum scores per subscale of the CTQ. **(A)** In AUD (left), females (red) reported significantly more severe CM for emotional and sexual abuse, and emotional neglect. **(B)** In CUD (right), females (red) reported significantly more severe CM for emotional and sexual abuse. CTQ, Childhood Trauma Questionnaire; EA, emotional abuse; PA, physical abuse, SA, sexual abuse, EN, emotional neglect; PN, physical neglect. Error bars are displayed at a 95% confidence interval. *Significant sex difference.

Over all substances, females reported more severe experiences of CM compared to men, resulting in significant sex differences for emotional abuse [*t*(242.3) = −4.14, *p* < 0.001] and sexual abuse [*t*(196.3) = −4.46, *p* < 0.001] (Figure 1). Sex differences regarding emotional neglect [*t*(628) = −2.16, *p* = 0.034] did not survive correction for multiple testing. Within each main diagnosis, significant sex differences to the detriment of women became apparent following two-sided *t*-tests for alcohol and emotional abuse [*t*(123.0) = −3.05, *p* = 0.003], alcohol and sexual abuse [*t*(100.75) = −2.77, *p* = 0.007], alcohol and emotional neglect [*t*(155.49) = −2.24, *p* = 0.026], cannabis and emotional abuse [*t*(46.50) = −3.31, *p* = 0.002] and cannabis and sexual abuse [*t*(33.40) = 2.54, *p* < 0.001]. Sex differences for physical neglect in individuals with CUD [*t*(38.22) = −2.24, *p* = 0.031] did not survive correction for multiple testing. See Figure 2 for more details.

### Severity of Childhood Maltreatment in Relation to Symptoms of Anxiety, Depressiveness, and Perceived Stress in the Overall Patient Sample

**FIGURE 3:**
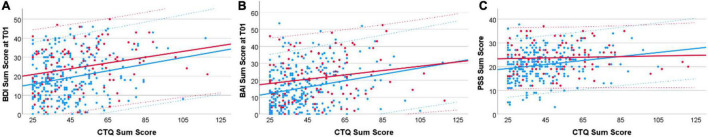
Correlation between CTQ sum score and **(A)** depressiveness (BDI), **(B)** anxiety (BAI), and **(C)** perceived stress (PSS). In males (blue), a significant positive correlation was observed for all three clinical variables. In women (red), a significant positive correlation was observed only for depressiveness. BAI, Beck Anxiety Inventory; BDI, Beck Depression Inventory; CTQ, Childhood Trauma Questionnaire; PSS, Perceived Stress Scale. Dotted lines indicate 95% confidence intervals.

Statistically significant positive correlations between the severity of CM (CTQ sum score) and affective symptoms were observed in the overall sample. See Figure 3 for more details. A positive correlation between the severity of CM and BDI sum score at admission was observed for males and females (males r = 0.241, *p* < 0.001; females r = 0.251, *p* = 0.012). The correlation between severity of CM and BAI sum score at admission and PSS sum score were significant for males (BAI r = 0.248, *p* < 0.001; PSS r = 0.207, *p* = 0.012), but not females (BAI r = 0.188, *p* = 0.062; PSS r = 0.044, *p* = 0.679). See Table 4 for more details.

**TABLE 4:** Severity of childhood maltreatment in relation to symptoms of anxiety, depressiveness, and perceived stress for the overall patient group, and separately by sex.

	BDI T01	BAI T01	PSS
	Corr. Coeff. | *p*-value | 1–β | *N*	Corr. Coeff. | *p*-value | 1–β | *N*	Corr. Coeff. | *p*-value | 1–β | *N*
**CTQ**			
All	**0.277 | <0.001 | >0.9999 | 391**	**0.259 | <0.001 | >0.9961 | 388**	**0.191 | <0.001 | >0.8393 | 351**
Males	**0.241 | <0.001 | >0.9023 | 291**	**0.248 | <0.001 | >0.8981 | 288**	**0.207 | <0.001 | >0.8641 | 261**
Females	**0.251 | <0.012 | >0.3254 | 100**	0.188 | >0.062 | – | 100	0.044 | <0.679 | – | 90

Pearson correlation coefficients, p-values (two-sided), and power estimates are displayed. Significant correlations are highlighted in bold. BAI, Beck Anxiety Inventory; BDI, Beck Depression Inventory; CTQ, Childhood Trauma Questionnaire, sum score; PSS, Perceived Stress Scale. All significant results survived correction for multiple testing (p > 0.05). Post hoc power calculations were performed in G*Power (57).

### The Influences of Different Types of Childhood Maltreatment on Substance Craving at Admission With Respect to Main Diagnosis and Sex

Craving at T01 (MACS T01) differed statistically significant for the different substance groups [*F*(4,381) = 2.622, *p* = 0.035, η^2^ = 0.027], and sex [*F*(1,381) = 6.771, *p* = 0.010, η^2^ = 0.017] after adjusting for all five subscores of the CTQ and age. Severity of emotional abuse [*F*(1,381) = 17.353, *p* < 0.001, η^2^ = 0.044] but none of the other subscales of CM or age did show a significant influence. After adjusting for before-mentioned covariates, Bonferroni-corrected *post hoc* tests revealed significantly more severe craving for women (*p* = 0.010, *M*_Diff_ = 2.92, 95% CI [0.71, 5.12]). *Post hoc* tests regarding substance group did not yield significant results following Bonferroni correction.

After adjusting for all five subscores of the CTQ and age but also PSS, BDI (T01) and BAI (T01) sum scores, craving at T01 (MACS T01) did no longer differ statistically significant between the different substance groups [*F*(4,282) = 2.516, *p* = 0.107, η^2^ = 0.027] or sex [*F*(1,282) = 2.516, *p* = 0.114, η^2^ = 0.009]. Severity of emotional abuse [*F*(1,282) = 1.282, *p* = 0.258, η^2^ = 0.005] did no longer show a significant influence, neither did the PSS sum score [*F*(1,282) = 0.735, *p* = 0.392, η^2^ = 0.003]. BDI and BAI sum scores at admission, however, did show a significant influence [*F*(1,282) = 43.637, *p* < 0.001, η^2^ = 0.134; *F*(1,282) = 15.360, *p* < 0.001, η^2^ = 0.052].

### The Influences of Different Types of Childhood Maltreatment on the Reduction of Substance Craving During the First 2 Weeks of Treatment With Respect to Main Diagnosis and Sex

Over all substances, craving diminished from 18.0 (10.0) at T01 to 11.0 (8.3) at T14 in the MACS questionnaire. However, no significant effect of substance group [*F*(4,306) = 0.836, *p* = 0.503, η^2^ = 0.011] or sex [*F*(1,306) = 3.516, *p* = 0.062, η^2^ = 0.011] was observed after adjusting for age and all five subscores of CM. There was no significant influence regarding all subscores of CM. Including PSS, BDI (T01) and BAI (T01), no significant effect of substance group [*F*(4,282) = 0.341, *p* = 0.850, η^2^ = 0.005] or sex [*F*(1,282) = 0.513, *p* = 0.475, η^2^ = 0.002] did emerge either. However, PSS and BDI (T01) sum scores excerpted a significant influence [*F*(1,282) = 14.433, *p* < 0.001, η^2^ = 0.049; *F*(1,282) = 21.050, *p* < 0.001, η^2^ = 0.069], so did age [*F*(1,282) = 5.095, *p* = 0.025, η^2^ = 0.018], but not the BAI (T01) sum score [*F*(1,282) = 2.807, *p* = 0.095, η^2^ = 0.010].

## Discussion

To our knowledge, this study is the first to examine a broad range of CM, namely emotional and physical abuse, emotional and physical neglect as well as sexual abuse in patients undergoing treatment for SUD while including several substances, such as alcohol, cannabis, cocaine and stimulant, opioid and sedative use disorders. The most salient finding of the present study was the high prevalence and severity of experienced CM in patients with CUD compared to other SUDs and especially compared to AUD. This study expands previous work on the relevance of psychosocial and biographical aspects regarding SUD.

The association between CM and SUD is well known in literature (12–21). The prevalence of moderate to extreme CM in our sample exceeded a previous estimation for the general German population ranging between 6.5% for emotional abuse and 22.4% for physical neglect (11). Similarly to the general population (11), women with SUD also reported higher prevalence rates for abuse but not neglect. Also, individuals with SUD suffered from significantly more severe experiences of CM for all subscales compared to the general German population (22). Our findings are consistent with previous studies, reporting a high prevalence and strong severity of CM in individuals with SUD (12, 15, 23, 58). Compared to a previous study on the severity of CM in individuals with SUD (23), we observed significantly less severe experiences of all forms of abuse but a more severe experience of physical neglect. A higher percentage of women in the previously reported SUD sample (41.3 vs. 27%) might contribute to these differences, since women are known to report higher severities of CM, which was also observed in our sample regarding emotional and sexual abuse. Also, Wingenfeld et al. (23) did not report on different substances. Depending on the composition of SUDs, group differences as we observed here might also contribute to the diverging observations.

Contrary to our hypothesis, individuals with OUD were not the most severely affected substance user group by CM in comparison to other SUD – although prevalence rates of OUD were comparable to previous studies (15, 58). This opposes previous research showing that individuals with OUD were more likely to report ACE in comparison to individuals with tobacco or cocaine use disorder (59). Others observed similar prevalence numbers of CM in both, individuals with OUD and matched controls, which was explained by the control group also containing individuals with other SUD. Still, males with OUD experienced significantly more physical and emotional abuse than controls, and females sexual abuse, respectively (60).

In our sample, patients with CUD showed both higher prevalence and more severe experiences of several subtypes of CM. Emotional abuse was significantly more severe in CUD compared to AUD. However, CUD compared to OUD did not reach significance. Individuals with CUD were similarly affected by comorbid mental disorders, i.e., schizophrenia, schizotypal and delusional disorders (F2), affective (F3), or neurotic, stress-related and somatoform disorders (F4) as AUD. *Post hoc* analyses (see Supplementary Material) for CUD and AUD did not yield significant group differences. However, individuals with CUD were diagnosed with more comorbid SUD compared to individuals with AUD. An explanation for our observation with respect to individuals with OUD might be three-fold. Firstly, an age effect cannot be ruled out regarding patients with CUD, since they were significantly younger. *Post hoc* analyses (see Supplementary Material) revealed a negative correlation between age and overall CM severity. However, within each substance group, including CUD, this correlation did not reach significance. Discussing generational aspect when it comes to (not) reporting CM are relevant, but beyond the scope of this retrospective, observational study. Secondly, CM data for OUD mainly derived from OMT patients. In contrast to the other SUD patients of our study, OMT patients were not abstinent, but continuously treated with opioids. Therefore the daily opioid treatment may have an acute effect and memories of CM might be suppressed to a certain extent. This could have led to an underreporting of prevalence and severity of CM. Opposing to this and besides psychobiological mechanism of withdrawal, in-house patients might find themselves strongly confronted with current problematic psychosocial factors during our treatment. They might increase attention toward traumatic events as one potential factor within the biopsychosocial model of addiction that is regularly discussed during medical and psychotherapeutic treatment of SUD. Thirdly, endocannabinoids mediate the extinction of aversive memories and regulate fear, anxiety and stress. External cannabis might enhance these effects, and thus might be consumed as a self-medication (61, 62). A systematic review of cannabis use motives identified negative life events, trauma, and maladaptive coping being related to consumption (63). This was also confirmed for CM as origin of negative stress and influenced by impairments in emotion regulation, e.g., negative mood (64). Cannabinoids are discussed as medical intervention for several anxiety- and trauma-related disorders by reason of their neuromodulator capacities in brain regions relevant for emotion and stress regulation (65, 66). Further research examined the hypothesis of a self-medication model of cannabis in posttraumatic stress disorder and revealed an acute, dose-dependent cannabis effect of a 51–67% symptom relief in more than 92% of cannabis users. However, a development of tolerance and therefore limited effects were observed (67).

Named considerations evoke the question of a causal origin of the association, namely whether CM is more frequent in SUD compared to the general population, because CM leads to SUD. Our analyses highlighted association between CM and SUD rather than causation. However, mechanisms identified in basic and animal research include a long lasting altered stress response after early life adversity. Further, perturbation of numerous neurodevelopmental processes, including the development and maturation of brain circuits involved in cognition and emotion, finally result in diminished cognitive control and increased desire for drug effects, i.e., memory extinction and relief from negative affect. Mechanisms are reviewed in Al’absi et al. (68) and Levis et al. (69). Recent basic research supported the contribution of CM to an increase in vulnerability for opioid addiction (Sophia C. (70)), possibly mediated by the endogenous opioid system which is involved in pro-social behavior in mammals, including humans (71). A recent review proposes “[…] based upon recent findings of opioid modulation of human social learning, bonding and empathy in relation to affiliative and protective tendencies. Fundamental to the model is that the mu-opioid system reinforces socially affiliative or protective behavior in response to positive and negative social experiences with long-term consequences for social behavior and health” (72). Lacking of pro-social touch, caring and protective behavior in childhood is a key feature of CM and may result in a long-term modification of the endogenous opioid system. On the emotional level this might result in an enhanced desire for social attachment and the pro-social effects of endogenous or external opioids. Not only opioids but all addictive substances share an activation of the opioid system, either by releasing endogenous opioids (alcohol, cannabis, amphetamines, and cocaine) or by direct activation of opioid receptors (heroin and synthetic opioids) (73–76). Therefore, this opioid pathway also increases the risk for non-opioid SUD in individuals having experienced CM.

In our sample, a positive relation between the severity of the overall CM and depressiveness, anxiety and perceived stress was observed for males. However, in women, current perceived stress did not relate to a history of CM. The relation between ACE such as CM and a later SUD has been observed to be party mediated by mood and anxiety disorders (33).

The influence of sex with regard to outcomes of CM has been discussed previously (77) and sex differences are commonly accepted. However, White and Kaffman (77) argued that despite similar presentation, underlying mechanisms might differ. Also, impairments in mental health following CM are subject to effects of gender and CM subtype (78). Potentially, physical abuse is more often related to internalizing mental disorders (e.g., affective disorders) in females subjects whereas in males physical abuse more often related to externalizing mental disorders (e.g., SUD) (79). For women, but not men, several subtypes of CM were associated with an increased risk for cocaine relapse (80). In cocaine, CM might increase the risk for relapses due to an increased appetitive anticipatory response to drug cues. Further, regulatory and control mechanism regarding stress- and cocaine-induces craving might be reduced following CM (81).

Substance craving refers to a multifaceted construct, including internal and external factors as well as corresponding interactions, that results in the desire or urge for consumption (82). Further, within the diagnosis of SUD, craving is listed as a relevant item (43). In our sample, substance craving at admission to treatment differed between sex and substance group and was influenced by emotional abuse, but not other types of CM. Higher craving at admission to SUD treatment was previously related to relapse, i.e., in individuals with AUD (35, 83), indicating the importance of monitoring craving and examining influencing factors. Regarding a diverging influence of specific subtypes of CM, physical and emotional abuse, as well as emotional neglect were previously associated with drug use (84) and emotional abuse, followed by physical abuse, were the strongest predictors for the severity of AUD (31). However, depressiveness as a current affective state exerted a strong influence on craving at admission and on craving reduction over the course of treatment. The influence of anxiety on craving became apparent only at admission, whereas perceived stress significantly contributed to craving reduction. Within our sample, a positive correlations between CM and symptoms of depressiveness, anxiety, and perceived stress have been observed. Individuals with CM are at higher risk for psychopathologies related to anxiety and depressiveness (4). At the same time, symptoms of depressiveness and anxiety are common for individuals entering treatment for SUD and negatively influence treatment outcome, i.e., increased risk for relapse (85). In AUD, inefficient emotion regulation is associated with increased alcohol craving and use (86). A history of CM was related to alcohol craving as a response to traumatic stimuli in healthy males. Further, physiological markers, such as cortisol reactivity, heart rate or skin conductance were also related to alcohol craving, CM or both (87).

### Limitations

Limitation, that might reduce the generalizability of the results have to be mentioned. First, possible limitations include the study being based on retrospective self-report questionnaires. Especially, when retrospectively assessing CM as it is done with the CTQ, answers might be biased. When assessing CM, a great heterogeneity regarding the instruments can be observed in the literature. Second, besides using questions defined by the authors, validated questionnaires, such as the CTQ, or interviews were used. When assessing ACE, CM has to be distinguished from a dysfunctional household (including divorce, substance use, observing intimate partner violence) *per se*. CM, abuse or neglect, account primarily for negative mental health outcomes in a study that examined individuals in their early and late adolescence (3). Due to the design of the here presented analyses, we did not assess other ACE besides CM as defined by CTQ and did not collect information about income or family structures which might have added to the biographical burden that possibly contributes to the development of SUD. This hinders the integration of study results in previous literature. Third, only patients were included in the analyses. Therefore, the influence of CM on the transition from low-risk to high-risk consumption possibly leading to a substance use disorder as well as characteristics inherent to non-treatment seeking individuals with SUD could not be examined. Fourth, substances were grouped and only the main diagnosis was considered. The small sample size for individuals with OUD or SHA does not allow for a broader discussion of the influence of main diagnosis on craving at admission and the reduction of craving during treatment.

### Clinical Implications

The here observed high prevalence and severity of CM in individuals with CUD, but also recent developments in the pattern of consumption and the potency of the available substances (88) underline the need for screening for CM both during treatment for CUD and in prevention of CUD. This is backed up by previous studies in individuals with both a history of CM and cannabis use that indicated a higher risk for psychotic symptoms in adolescents (89) and a more severe symptomatology for bipolar disorder (90). Irrespective of the substance of use, a high prevalence and severity of CM was underlining the importance of assessing CM with suitable tools in all settings of SUD prevention and treatment. If CM can be ceased and a positive environment is installed including intact social networks, positive coping, self-esteem and optimism, the neuro-adaptive capacities of the human brain might allow for a positive outcome, even following CM (91). For example, low levels of mindfulness might link CM to alcohol use (92), therefore serving as a therapeutic target. Individuals with SUD and CM might benefit from integrative psychosocial interventions targeting both, trauma-related and SUD-related symptoms (93), such as interpersonal psychotherapy (94) or trauma informed yoga (95, 96).

## Conclusion

Individuals with SUD experience various forms of CM more often and in a more severe manner than the general population. SUD group differences with regard to prevalence and severity of CM were observed. Sex differences to the detriment of women can be observed in several SUDs. CM, specifically emotional abuse, might be related to craving at admission to treatment. However, pathways of mediating factors, such as depressiveness, anxiety and stress still have to be examined in more depth. Also, underlying causal and explanatory mechanism such as impairments in processing of trauma history, emotional regulation, or neurobiological alterations following CM remain to be further examined. A history of CM should be assessed during treatment for SUD. A possible positive influence of trauma-related interventions during SUD treatment specifically addressing aspects of CM on treatment outcomes and relapse rates can be hypothesized.

## Data Availability Statement

Data contains sensitive medical information and will be made available for researchers who meet the criteria for access to confidential data. The data underlying the results presented in the study are available upon reasonable request from SV-K (s.vollstaedt-klein@zi-mannheim.de).

## Ethics Statement

The studies involving human participants were reviewed and approved by the Local Ethics Committee of the Medical Faculty Mannheim, Heidelberg University, Mannheim, Germany (approval numbers 2018-531N-MA and 2018-807R-MA). The patients/participants provided their written informed consent to participate in this study.

## Author Contributions

SG designed the current study, performed the data analysis, and drafted the manuscript. KE and NB collected the parts of the data from study 2. SH supported the data analysis. KE, DH, AK, WS, and SV-K contributed to the interpretation of the data. KE helped with the writing of the manuscript. SV-K, FK, PB, NB, DH, AK, and WS were responsible for the study designs of the original studies and helped with the recruitment. DH, FK, and WS procured the funding of the original studies. All authors revised the manuscript critically for important intellectual content and approved the final version.

## Conflict of Interest

DH was employed by the Therapieverbund Ludwigsmühle gGmbH. The remaining authors declare that the research was conducted in the absence of any commercial or financial relationships that could be construed as a potential conflict of interest.

## Publisher’s Note

All claims expressed in this article are solely those of the authors and do not necessarily represent those of their affiliated organizations, or those of the publisher, the editors and the reviewers. Any product that may be evaluated in this article, or claim that may be made by its manufacturer, is not guaranteed or endorsed by the publisher.

## References

[1] Gilbert R Widom CS Browne K Fergusson D Webb E Janson S. Burden and consequences of child maltreatment in high-income countries. *Lancet.* (2009) 373:68–81. 10.1016/S0140-6736(08)61706-7 19056114

[2] Brown DW Anda RF Tiemeier H Felitti VJ Edwards VJ Croft JB Adverse childhood experiences and the risk of premature mortality. *Am J Prevent Med.* (2009) 37:389–96. 10.1016/j.amepre.2009.06.021 19840693

[3] Negriff S. ACEs are not equal: examining the relative impact of household dysfunction versus childhood maltreatment on mental health in adolescence. *Soc Sci Med.* (2020) 245:112696. 10.1016/j.socscimed.2019.112696 31785426 PMC6961803

[4] Teicher MH Samson JA. Childhood maltreatment and psychopathology: a case for ecophenotypic variants as clinically and neurobiologically distinct subtypes. *Am J Psychiatry.* (2013) 170:1114–33. 10.1176/appi.ajp.2013.12070957 23982148 PMC3928064

[5] Heim C Binder EB. Current research trends in early life stress and depression: review of human studies on sensitive periods, gene-environment interactions, and epigenetics. *Exp Neurol.* (2012) 233:102–11. 10.1016/j.expneurol.2011.10.032 22101006

[6] Hughes K Lowey H Quigg Z Bellis MA. Relationships between adverse childhood experiences and adult mental well-being: results from an English national household survey. *BMC Public Health.* (2016) 16:222. 10.1186/s12889-016-2906-3 26940088 PMC4778324

[7] Hussey JM Chang JJ Kotch JB. Child maltreatment in the United States: prevalence, risk factors, and adolescent health consequences. *Pediatrics.* (2006) 118:933–42. 10.1542/peds.2005-2452 16950983

[8] Moustafa AA Parkes D Fitzgerald L Underhill D Garami J Levy-Gigi E The relationship between childhood trauma, early-life stress, and alcohol and drug use, abuse, and addiction: an integrative review. *Curr Psychology.* (2021) 40:579–84. 10.1007/s12144-018-9973-9

[9] Raabe FJ Spengler D. Epigenetic risk factors in PTSD and depression. *Front Psychiatry.* (2013) 4:80. 10.3389/fpsyt.2013.00080 23966957 PMC3736070

[10] Sethi D Bellis M Hughes K Gilbert R Mitis F Galea G. *European Report on Preventing Child Maltreatment.* Copenhagen: World Health Organization (2013).

[11] Witt A Brown RC Plener PL Brähler E Fegert JM. Child maltreatment in Germany: prevalence rates in the general population. *Child Adolesc Psychiatr Ment Health.* (2017) 11:47. 10.1186/s13034-017-0185-0 28974983 PMC5621113

[12] Choi NG DiNitto DM Marti CN Choi BY. Association of adverse childhood experiences with lifetime mental and substance use disorders among men and women aged 50+ years. *Int Psychogeriatrics.* (2016) 29:359–72. 10.1017/S1041610216001800 27780491

[13] Cutajar MC Mullen PE Ogloff JR Thomas SD Wells DL Spataro J. Psychopathology in a large cohort of sexually abused children followed up to 43 years. *Child Abuse Negl.* (2010) 34:813–22. 10.1016/j.chiabu.2010.04.004 20888636

[14] Hägele C Friedel E Kienast T Kiefer F. How do we ‘learn’ addiction? Risk factors and mechanisms getting addicted to alcohol. *Neuropsychobiology.* (2014) 70:67–76. 10.1159/000364825 25359487

[15] Santo T Campbell G Gisev N Tran LT Colledge S Di Tanna GL Prevalence of childhood maltreatment among people with opioid use disorder: a systematic review and meta-analysis. *Drug Alcohol Depend.* (2021) 219:108459. 10.1016/j.drugalcdep.2020.108459 33401031 PMC7855829

[16] Schafer I Pawils S Driessen M Harter M Hillemacher T Klein M Understanding the role of childhood abuse and neglect as a cause and consequence of substance abuse: the German CANSAS network. *Eur J Psychotraumatol.* (2017) 8:1304114. 10.1080/20008198.2017.1304114 28451071 PMC5399994

[17] Afifi TO Taillieu T Salmon S Davila IG Stewart-Tufescu A Fortier J Adverse childhood experiences (ACEs), peer victimization, and substance use among adolescents. *Child Abuse Negl.* (2020) 106:104504. 10.1016/j.chiabu.2020.104504 32402816

[18] Anda RF Felitti VJ Bremner JD Walker JD Whitfield C Perry BD The enduring effects of abuse and related adverse experiences in childhood. A convergence of evidence from neurobiology and epidemiology. *Eur Arch Psychiatry Clin Neurosci.* (2006) 256:174–86. 10.1007/s00406-005-0624-4 16311898 PMC3232061

[19] Kirsch D Nemeroff CM Lippard ETC. Early life stress and substance use disorders: underlying neurobiology and pathways to adverse outcomes. *Advers Resil Sci.* (2020) 1:29–47. 10.1007/s42844-020-00005-7 PMC11995910 40235604

[20] Felsher JR Derevensky JL Gupta R. Young adults with gambling problems: the impact of childhood maltreatment. *Int J Ment Health Addict.* (2010) 8:545–56. 10.1007/s11469-009-9230-4

[21] Poole JC Kim HS Dobson KS Hodgins DC. Adverse childhood experiences and disordered gambling: assessing the mediating role of emotion dysregulation. *J Gambl Stud.* (2017) 33:1187–200. 10.1007/s10899-017-9680-8 28258336

[22] Klinitzke G Romppel M Hauser W Brahler E Glaesmer H. [The German Version of the Childhood Trauma Questionnaire (CTQ): psychometric characteristics in a representative sample of the general population]. *Psychother Psychosom Med Psychol.* (2012) 62:47–51. 10.1055/s-0031-1295495 22203470

[23] Wingenfeld K Spitzer C Mensebach C Grabe HJ Hill A Gast U [The German version of the childhood trauma questionnaire (CTQ): preliminary psychometric properties]. *Psychother Psychosom Med Psychol.* (2010) 60:442–50. 10.1055/s-0030-1247564 20200804

[24] Andersson HW Wenaas M Nordfjærn T. Relapse after inpatient substance use treatment: a prospective cohort study among users of illicit substances. *Addict Behav.* (2019) 90:222–8. 10.1016/j.addbeh.2018.11.008 30447514

[25] Moos RH Moos BS. Rates and predictors of relapse after natural and treated remission from alcohol use disorders. *Addiction.* (2006) 101:212–22. 10.1111/j.1360-0443.2006.01310.x 16445550 PMC1976118

[26] Peacock A Leung J Larney S Colledge S Hickman M Rehm J Global statistics on alcohol, tobacco and illicit drug use: 2017 status report. *Addiction.* (2018) 113:1905–26. 10.1111/add.14234 29749059

[27] Afifi TO Henriksen CA Asmundson GJ Sareen J. Childhood maltreatment and substance use disorders among men and women in a nationally representative sample. *Can J Psychiatry.* (2012) 57:677–86. 10.1177/070674371205701105 23149283

[28] Oberleitner LM Smith PH Weinberger AH Mazure CM McKee SA. Impact of exposure to childhood maltreatment on transitions to alcohol dependence in women and men. *Child Maltreat.* (2015) 20:301–8. 10.1177/1077559515591270 26130105 PMC4868049

[29] Turner RJ Lloyd DA. Cumulative adversity and drug dependence in young adults: racial/ethnic contrasts. *Addiction.* (2003) 98:305–15. 10.1046/j.1360-0443.2003.00312.x 12603230

[30] Alvanzo AAH Storr CL Reboussin B Green KM Mojtabai R La Flair LN Adverse childhood experiences (ACEs) and transitions in stages of alcohol involvement among US adults: progression and regression. *Child Abuse Negl.*(2020) 107:104624. 10.1016/j.chiabu.2020.104624 32683202 PMC7968748

[31] Schwandt ML Heilig M Hommer DW George DT Ramchandani VA. Childhood trauma exposure and alcohol dependence severity in adulthood: mediation by emotional abuse severity and neuroticism. *Alcohol Clin Exp Res.* (2013) 37:984–92. 10.1111/acer.12053 23278300 PMC3620963

[32] Schückher F Sellin T Fahlke C Engström I. The impact of childhood maltreatment on age of onset of alcohol use disorder in women. *Eur Addict Res.* (2018) 24:278–85. 10.1159/000494766 30448841

[33] Douglas KR Chan G Gelernter J Arias AJ Anton RF Weiss RD Adverse childhood events as risk factors for substance dependence: partial mediation by mood and anxiety disorders. *Addict Behav.* (2010) 35:7–13. 10.1016/j.addbeh.2009.07.004 19720467 PMC2763992

[34] Paliwal P Hyman SM Sinha R. Craving predicts time to cocaine relapse: further validation of the now and brief versions of the cocaine craving questionnaire. *Drug Alcohol Depend.* (2008) 93:252–9. 10.1016/j.drugalcdep.2007.10.002 18063320 PMC2254317

[35] Schneekloth TD Biernacka JM Hall-Flavin DK Karpyak VM Frye MA Loukianova LL Alcohol craving as a predictor of relapse. *Am J Addict.* (2012) 21(Suppl. 1):S20–6. 10.1111/j.1521-0391.2012.00297.x 23786506

[36] Stohs ME Schneekloth TD Geske JR Biernacka JM Karpyak VM. Alcohol craving predicts relapse after residential addiction treatment. *Alcohol Alcohol.* (2019) 54:167–72. 10.1093/alcalc/agy093 30796778

[37] Tsui JI Anderson BJ Strong DR Stein MD. Craving predicts opioid use in opioid-dependent patients initiating buprenorphine treatment: a longitudinal study. *Am J Drug Alcohol Abuse.* (2014) 40:163–9. 10.3109/00952990.2013.848875 24521036 PMC4868341

[38] Ilgen M Jain A Kim HM Trafton JA. The effect of stress on craving for methadone depends on the timing of last methadone dose. *Behav Res Ther.* (2008) 46:1170–5. 10.1016/j.brat.2008.05.013 18675399

[39] Sinha R Catapano D O’Malley S. Stress-induced craving and stress response in cocaine dependent individuals. *Psychopharmacology.* (1999) 142:343–51. 10.1007/s002130050898 10229058

[40] Clay JM Adams C Archer P English M Hyde A Stafford LD Psychosocial stress increases craving for alcohol in social drinkers: effects of risk-taking. *Drug Alcohol Depend.* (2018) 185:192–7. 10.1016/j.drugalcdep.2017.12.021 29462766

[41] Berhe O Gerhardt S Schmahl C. Clinical outcomes of severe forms of early social stress. *Curr Top Behav Neurosci.* (2021). 10.1007/7854_2021_261 [Epub ahead of print]. 34628586

[42] Mann K Loeber S Croissant B Kiefer F. Qualifizierte Entzugsbehandlung von Alkoholabhängigen. Ein Manual zur Pharmako- und Psychotherapie. *Dtsch Arztebl Int.* (2006) 103:3252.

[43] American Psychiatric Association. *Diagnostic and Statistical Manual of Mental Disorders: DSM-5™.* 5th ed. Arlington, VA: American Psychiatric Publishing, Inc (2013).

[44] Dawson DA Goldstein RB Grant BF. Differences in the profiles of DSM-IV and DSM-5 alcohol use disorders: implications for clinicians. *Alcoholism Clin Exp Res.* (2013) 37:E305–13. 10.1111/j.1530-0277.2012.01930.x 22974144 PMC3800556

[45] Eidenmueller K Grimm F Hermann D Frischknecht U Montag C Dziobek I Exploring influences on theory of mind impairment in opioid dependent patients. *Front Psychiatry.* (2021) 12:721690. 10.3389/fpsyt.2021.721690 34887783 PMC8649648

[46] Bernstein DP Stein JA Newcomb MD Walker E Pogge D Ahluvalia T Development and validation of a brief screening version of the Childhood Trauma Questionnaire. *Child Abuse Negl.* (2003) 27:169–90. 10.1016/S0145-2134(02)00541-0 12615092

[47] Bernstein DP Fink L Handelsman L Foote J Lovejoy M Wenzel K Initial reliability and validity of a new retrospective measure of child abuse and neglect. *Am J Psychiatry.* (1994) 151:1132–6. 10.1176/ajp.151.8.1132 8037246

[48] Häuser W Schmutzer G Brähler E Glaesmer H. Misshandlungen in Kindheit und Jugend. *Dtsch Arztebl Int.* (2011) 108:287–94. 10.3238/arztebl.2011.0287 21629512 PMC3103979

[49] Cohen S Kamarck T Mermelstein R. A global measure of perceived stress. *J Health Soc Behav.* (1983) 24:385–96. 10.2307/2136404 6668417

[50] Heatherton TF Kozlowski LT Frecker RC Fagerstrom KO. The fagerstrom test for nicotine dependence: a revision of the fagerstrom tolerance questionnaire. *Br J Addict.* (1991) 86:1119–27. 10.1111/j.1360-0443.1991.tb01879.x 1932883

[51] Beck AT Ward C Mendelson M Mock J Erbaugh J. Beck depression inventory (BDI). *Arch Gen Psychiatry.* (1961) 4:561–71. 13688369 10.1001/archpsyc.1961.01710120031004

[52] Kühner C Bürger C Keller F Hautzinger M. [Reliability and validity of the revised beck depression inventory (BDI-II). results from german samples]. *Nervenarzt.* (2007) 78:651–6. 10.1007/s00115-006-2098-7 16832698

[53] Beck AT Epstein N Brown G Steer RA. An inventory for measuring clinical anxiety: psychometric properties. *J Consult Clin Psychol.* (1988) 56:893–7. 10.1037//0022-006x.56.6.893 3204199

[54] Nakovics H Diehl A Geiselhart H Mann K. [Development and validation of an overall instrument to measure craving across multiple substances: the Mannheimer craving scale (MaCS)]. *Psychiatr Prax.* (2009) 36:72–8. 10.1055/s-2008-1067546 18924060

[55] García-pérez MA Núñez-antón V. Cellwise residual analysis in two-way contingency tables. *Educ Psychol Meas.* (2003) 63:825–39. 10.1177/0013164403251280

[56] Storey JD. A direct approach to false discovery rates. *J R Stat Soc.* (2002) 64:479–98. 10.1111/1467-9868.00346

[57] Faul F Erdfelder E Lang A-G Buchner A. G*Power 3: a flexible statistical power analysis program for the social, behavioral, and biomedical sciences. *Behav Res Methods.* (2007) 39:175–91. 10.3758/BF03193146 17695343

[58] Medrano MA Zule WA Hatch J Desmond DP. Prevalence of childhood trauma in a community sample of substance-abusing women. *Am J Drug Alcohol Abuse.* (1999) 25:449–62. 10.1081/ADA-100101872 10473008

[59] Lawson KM Back SE Hartwell KJ Maria MMS Brady KT. A comparison of trauma profiles among individuals with prescription opioid, nicotine, or cocaine dependence. *Am J Addict.* (2013) 22:127–31. 10.1111/j.1521-0391.2013.00319.x 23414497 PMC3681508

[60] Conroy E Degenhardt L Mattick RP Nelson EC. Child maltreatment as a risk factor for opioid dependence: comparison of family characteristics and type and severity of child maltreatment with a matched control group. *Child Abuse Negl.* (2009) 33:343–52. 10.1016/j.chiabu.2008.09.009 19477004 PMC2729332

[61] Lutz B Marsicano G Maldonado R Hillard CJ. The endocannabinoid system in guarding against fear, anxiety and stress. *Nat Rev Neurosci.* (2015) 16:705–18. 10.1038/nrn4036 26585799 PMC5871913

[62] Marsicano G Wotjak CT Azad SC Bisogno T Rammes G Cascio MG The endogenous cannabinoid system controls extinction of aversive memories. *Nature.* (2002) 418:530–4. 10.1038/nature00839 12152079

[63] Hyman SM Sinha R. Stress-related factors in cannabis use and misuse: implications for prevention and treatment. *J Subst Abuse Treat.* (2009) 36:400–13. 10.1016/j.jsat.2008.08.005 19004601 PMC2696937

[64] Vilhena-Churchill N Goldstein AL. Child maltreatment and marijuana problems in young adults: examining the role of motives and emotion dysregulation. *Child Abuse Negl.* (2014) 38:962–72. 10.1016/j.chiabu.2013.10.009 24268374

[65] Kondev V Winters N Patel S. Chapter Four – cannabis use and posttraumatic stress disorder comorbidity: epidemiology, biology and the potential for novel treatment approaches. In: Calipari ES Gilpin NW editors. *International Review of Neurobiology.* (Vol. 157), Cambridge, MA: Academic Press (2021). p. 143–93. 10.1016/bs.irn.2020.09.007 33648669

[66] Papagianni EP Stevenson CW. Cannabinoid regulation of fear and anxiety: an update. *Curr psychiatry Rep.* (2019) 21:38. 10.1007/s11920-019-1026-z 31030284 PMC6486906

[67] LaFrance EM Glodosky NC Bonn-Miller M Cuttler C. Short and long-term effects of cannabis on symptoms of post-traumatic stress disorder. *J Affect Disord.* (2020) 274:298–304. 10.1016/j.jad.2020.05.132 32469819

[68] Al’absi M Ginty AT Lovallo WR. Neurobiological mechanisms of early life adversity, blunted stress reactivity and risk for addiction. *Neuropharmacology.* (2021) 188:108519. 10.1016/j.neuropharm.2021.108519 33711348 PMC9195251

[69] Levis SC Baram TZ Mahler SV. Neurodevelopmental origins of substance use disorders: evidence from animal models of early-life adversity and addiction. *Eur J Neurosci.* (2021) 1–26. 10.1111/ejn.15223 33825217 PMC8494863

[70] Levis SC Bentzley BS Molet J Bolton JL Perrone CR Baram TZ On the early life origins of vulnerability to opioid addiction. *Mol Psychiatry.* (2021) 26:4409–16. 10.1038/s41380-019-0628-5 31822817 PMC7282971

[71] Loseth G Ellingsen D-M Leknes S. State-dependent μ-opioid modulation of social motivation – a model. *Front Behav Neurosci.* (2014) 8:430. 10.3389/fnbeh.2014.00430 25565999 PMC4264475

[72] Meier IM van Honk J Bos PA Terburg D. A mu-opioid feedback model of human social behavior. *Neurosci Biobehav Rev.* (2021) 121:250–8. 10.1016/j.neubiorev.2020.12.013 33359094

[73] Charbogne P Kieffer BL Befort K. 15 years of genetic approaches in vivo for addiction research: opioid receptor and peptide gene knockout in mouse models of drug abuse. *Neuropharmacology.* (2014) 76(Pt B):204–17. 10.1016/j.neuropharm.2013.08.028 24035914 PMC3858501

[74] Mitchell JM O’Neil JP Janabi M Marks SM Jagust WJ Fields HL. Alcohol consumption induces endogenous opioid release in the human orbitofrontal cortex and nucleus accumbens. *Sci Transl Med.* (2012) 4:116ra6. 10.1126/scitranslmed.3002902 22238334

[75] Olive MF Koenig HN Nannini MA Hodge CW. Stimulation of endorphin neurotransmission in the nucleus accumbens by ethanol, cocaine, and amphetamine. *J Neurosci.* (2001) 21:Rc184. 10.1523/JNEUROSCI.21-23-j0002.2001 11717387 PMC6763912

[76] Valverde O Noble F Beslot F Daugé V Fournié-Zaluski MC Roques BP. Delta9-tetrahydrocannabinol releases and facilitates the effects of endogenous enkephalins: reduction in morphine withdrawal syndrome without change in rewarding effect. *Eur J Neurosci.* (2001) 13:1816–24. 10.1046/j.0953-816x.2001.01558.x 11359533

[77] White JD Kaffman A. The moderating effects of sex on consequences of childhood maltreatment: from clinical studies to animal models. *Front Neurosci.* (2019) 13:1082. 10.3389/fnins.2019.01082 31680821 PMC6797834

[78] Rehan W Antfolk J Johansson A Jern P Santtila P. Experiences of severe childhood maltreatment, depression, anxiety and alcohol abuse among adults in Finland. *PLoS One.* (2017) 12:e0177252. 10.1371/journal.pone.0177252 28481912 PMC5421798

[79] Keyes KM Eaton NR Krueger RF McLaughlin KA Wall MM Grant BF Childhood maltreatment and the structure of common psychiatric disorders. *Br J Psychiatry.* (2012) 200:107–15. 10.1192/bjp.bp.111.093062 22157798 PMC3269653

[80] Hyman SM Paliwal P Chaplin TM Mazure CM Rounsaville BJ Sinha R. Severity of childhood trauma is predictive of cocaine relapse outcomes in women but not men. *Drug Alcohol Depend.* (2008) 92:208–16. 10.1016/j.drugalcdep.2007.08.006 17900822 PMC2233653

[81] Elton A Smitherman S Young J Kilts CD. Effects of childhood maltreatment on the neural correlates of stress- and drug cue-induced cocaine craving. *Addict Biol.* (2015) 20:820–31. 10.1111/adb.12162 25214317 PMC4362751

[82] Tiffany ST Wray JM. The clinical significance of drug craving. *Ann N Y Acad Sci.* (2012) 1248:1–17. 10.1111/j.1749-6632.2011.06298.x 22172057 PMC4041083

[83] Bottlender M Soyka M. Impact of craving on alcohol relapse during, and 12 months following, outpatient treatment. *Alcohol Alcohol.* (2004) 39:357–61. 10.1093/alcalc/agh073 15208171

[84] Norman RE Byambaa M De R Butchart A Scott J Vos T. The long-term health consequences of child physical abuse, emotional abuse, and neglect: a systematic review and meta-analysis. *PLoS Med.* (2012) 9:e1001349. 10.1371/journal.pmed.1001349 23209385 PMC3507962

[85] Charney DA Palacios-Boix J Negrete JC Dobkin PL Gill KJ. Association between concurrent depression and anxiety and six-month outcome of addiction treatment. *Psychiatr Serv.* (2005) 56:927–33. 10.1176/appi.ps.56.8.927 16088008

[86] Petit G Luminet O Maurage F Tecco J Lechantre S Ferauge M Emotion regulation in alcohol dependence. *Alcohol Clin Exp Res.* (2015) 39:2471–9. 10.1111/acer.12914 26613633

[87] Trautmann S Kraplin A Dieterich R Richter J Muehlhan M. The role of childhood trauma and stress reactivity for increased alcohol craving after induced psychological trauma: an experimental analogue study. *Psychopharmacology.* (2018) 235:2883–95. 10.1007/s00213-018-4979-4 30203300

[88] Manthey J. Cannabis use in Europe: current trends and public health concerns. *Int J Drug Policy.* (2019) 68:93–6. 10.1016/j.drugpo.2019.03.006 31030057

[89] Harley M Kelleher I Clarke M Lynch F Arseneault L Connor D Cannabis use and childhood trauma interact additively to increase the risk of psychotic symptoms in adolescence. *Psychol Med.* (2010) 40:1627–34. 10.1017/S0033291709991966 19995476

[90] Aas M Etain B Bellivier F Henry C Lagerberg T Ringen A Additive effects of childhood abuse and cannabis abuse on clinical expressions of bipolar disorders. *Psychol Med.* (2014) 44:1653–62. 10.1017/S0033291713002316 24028906

[91] Holz NE Tost H Meyer-Lindenberg A. Resilience and the brain: a key role for regulatory circuits linked to social stress and support. *Mol Psychiatry.* (2020) 25:379–96. 10.1038/s41380-019-0551-9 31628419

[92] Brett EI Espeleta HC Lopez SV Leavens ELS Leffingwell TR. Mindfulness as a mediator of the association between adverse childhood experiences and alcohol use and consequences. *Addict Behav.* (2018) 84:92–8. 10.1016/j.addbeh.2018.04.002 29653433

[93] Brady KT Back SE. Childhood trauma, posttraumatic stress disorder, and alcohol dependence. *Alcohol Res.* (2012) 34:408–13. 23584107 10.35946/arcr.v34.4.05 PMC3860395

[94] Markowitz JC Kocsis JH Christos P Bleiberg K Carlin A. Pilot Study of interpersonal psychotherapy versus supportive psychotherapy for dysthymic patients with secondary alcohol abuse or dependence. *J Nerv Ment Dis.* (2008) 196:468–74. 10.1097/NMD.0b013e31817738f1 18552624

[95] Esfeld J Pennings K Rooney A Robinson S. Integrating trauma-informed yoga into addiction treatment. *J Creat Ment Health.* (2021) 1–10. 10.1080/15401383.2021.1972067

[96] Macy RJ Jones E Graham LM Roach L. Yoga for trauma and related mental health problems: a meta-review with clinical and service recommendations. *Trauma Violence Abuse.* (2018) 19:35–57. 10.1177/1524838015620834 26656487

[97] Heinz A Kiefer F Smolka MN Endrass T Beste C Beck A Addiction research consortium: losing and regaining control over drug intake (ReCoDe)-from trajectories to mechanisms and interventions. *Addict Biol*. (2020) 25:e12866. 10.1111/adb.12866 31859437

