# Supplementary material for: A History of Childhood Maltreatment Has Substance- and Sex-Specific Effects on Craving During Treatment for Substance Use Disorders
Source: Front Psychiatry. 2022 Apr 14;13:866019. doi: 10.3389/fpsyt.2022.866019 (PMC9046680; doi:10.3389/fpsyt.2022.866019)
Supplement: Supplementary file 1 [file Data_Sheet_1.docx]

**A History of Childhood Maltreatment Has Substance- and Sex-Specific Effects on Craving During Treatment for Substance Use Disorder**

**Supplementary Material**

1. **Flow-chart of data collection and preparation procedure**

Patients from the Clinic of Addictive Behaviour and Addiction Medicine

- n = 1483

Study participants from Outpatient Clinic for Substitution Medicine

- n =116

## Data collection

## Data preparation (n=1599)

Excluded (n=944)

- No CTQ*
- Duplicate cases/ readmission**
- Main diagnosis other than SUD***

## Allocation (n=655)

## AUD

***n=364***

## CUD

***n=118***

## OUD

***n=9 +109***

## SHA

***n=12***

## CSUD

***n=43***

Note: n = sample size; AUD = Alcohol Use Disorder; CTQ = Childhood Trauma Questionnaire; CUD = Cannabis Use Disorder; OUD = Opioid Use Disorder (N = 9) including Opioid Maintenance Treatment (OUT, N = 109); SHA = Sedative, Hypnotics, or Anxiolytic Use Disorders; SUD = Substance Use Disorder

* Only individuals with at least one CTQ sub score were included, individuals without CTQ data were excluded (N = 794)

** The most recent dataset was used for patients with readmissions over the 5-year observation period, data results from repeated admissions was excluded (N = 78)

*** Only individuals with the main diagnosis of SUD were included. Other main diagnoses, such as personality disorders or other mental disorders were excluded from subsequent analyses (N = 72).

1. **Supplementary Analyses**

**Comparison of individuals with opioid use disorder (OUD) between sample 1 and sample 2**

Patients with OSUD (sample 1) and OMT (sample 2) did not differ in CTQ subscores, the dichotomous variable ‘having experienced childhood maltreatment (CM), yes or no’, and the total number of CM, as well as age, sex, family status, having children or employment status (p > .1, t-test 2-tailed; p > .05, chi-square tests). Because the two samples did not differ for the main sociodemographic variables sex, age and CTQ subscores, both samples were merged for subsequent analyses regarding hypotheses one and two.

**Correlation between CM and age**

Correlation analyses between the severity of CM (CTQ sum score) and age revealed a significant association for the overall sample while controlling for sex (r = -0.169 p = 0.004). However, within each substance, this sex-controlled correlation did not reach significance (AUD (r = -0.037, p = 0.633), CUD (r = -0.024, p = 0.838), CSUD (r = 0.101, p =0.655), SHA (r = -0.043, p = 0.957), OUD (r = 0.473, p = 0.421).

**Supplementary Table 1: Comorbidities in AUD and CUD.** Mean values (standard deviation) or percentage values are displayed

|  | **AUD** | **CUD** | **Descriptive Statistics** |
| --- | --- | --- | --- |
| **N** | 364 | 118 |  |
| **F2 (% yes)** | 1.1 | 2.5 | χ²(1) = 1.288, p = .256 |
| **F3 - current (% yes)** | 33.5 | 33.9 | χ²(1) = 0.003, p = .954 |
| **F3 - lifetime (% yes)** | 39.3 | 37.3 | χ²(1) = 0.150, p = .699 |
| **F4 – current (% yes)** | 13.5 | 12.7 | χ²(1) = 0.048, p = .827 |
| **F4 – lifetime (% yes)** | 22.8 | 15.3 | χ²(1) = 3.065, p = .080 |

**Note**: n = total sample size; AUD = Alcohol Use Disorder; CUD = Cannabis Use Disorder; F2 = Schizophrenia, Schizotypal and Delusional Disorders; F3 = Mood (Affective) Disorders; F4 = Neurotic, Stress-related and Somatoform Disorders.

**Severity of different subtypes of CM in patients with SUD compared to the general population in Germany and a previous sample of individuals with SUD**

To compare the severity of CM in the present sample with a representative sample of the German population ([Klinitzke et al., 2012](#_ENREF_1)), one sample t-tests were performed for all subscales of the CTQ separately, including the respective mean from the population-based study as test value. Similarly, the here presented data was compared to a German sample with SUD ([Wingenfeld et al., 2010](#_ENREF_2)). Individuals with SUD reported higher severity for all subscales of CM compared to a German representative sample ([Klinitzke et al., 2012](#_ENREF_1)), i.e., emotional abuse (t(626) = 16.374, p < 0.001), physical abuse (t(618) = 10.394, p < 0.001), sexual abuse (t(621) = 5.639, p < 0.001); emotional neglect (t(629) = 13.882, p < 0.001) and physical neglect (t(631) = 4.946, p < 0.001). Further, comparing the severity of CM to a previous study on SUD ([Wingenfeld et al., 2010](#_ENREF_2)) our sample was less affected by emotional abuse (t(626) = -4.013, p < 0.001), physical abuse ((618) = -6.842, p < 0.001), and sexual abuse (t(621) = -3.905, p < 0.001), and more affected by physical neglect (t(631) = 2.122, p = 0.034). Emotional neglect did not yield significant differences (t(629) = 0.875, p = 0.382).

**The influence of different types of CM on substance craving at admission**

Craving at T01 (MACS T01) differed statistically significant for sex (F(1, 370) = 6.706, p = 0.010, η² = 0.018) but not the different substance groups (F(2, 370) = 3.027, p = 0.050, η² = 0.016) after adjusting for all five subscores of the CTQ and age. Emotional abuse (F(1, 370) = 16.482, p < 0.001, η² = 0.043) but none of the other subscales of CM or age did show a significant influence. After adjusting for before-mentioned covariates, Bonferroni-corrected post-hoc tests revealed significantly more severe craving for women (p = 0.010, M_Diff_ = 2.98, 95%-CI[0.72, 5.25]). Post-hoc tests regarding substance group revealed significantly more severe craving for CSUD compared to AUD (p = 0.043, M_Diff_ = 4.40, 95%-CI[0.093, 8.70]).

After adjusting for all five subscores of the CTQ and age but also PSS, BDI (T01) and BAI (T01) sum scores, craving at T01 (MACS T01) differed statistically significant between the different substance groups (F(2, 272) = 3.990, p = 0.020, η² = 0.029) and sex (F(1, 272) = 4.095, p = 0.044, η² = 0.015). Emotional abuse (F(1, 272) = 0.508, p = 0.477, η² = 0.002) did no longer show a significant influence, neither did the PSS sum score (F(1, 272) = 0.782, p = 0.377, η² = 0.003). BDI and BAI sum scores at admission, however, did show a significant influence (F(1, 272) = 47.825, p < 0.001, η² = 0.150; F(1, 272) = 17.697, p < 0.001, η² = 0.061). After adjusting for before-mentioned covariates, Bonferroni-corrected post-hoc tests revealed significantly more severe craving for women (p = 0.044, M_Diff_ = 2.10, 95%-CI[0.06, 4.14]). Post-hoc tests regarding substance group revealed significantly more severe craving for CSUD compared to AUD (p = 0.025, M_Diff_ = 4.35, 95%-CI[0.41, 8.30]) and CUD (p = 0.029, M_Diff_ = 4.39, 95%-CI[0.34, 8.44]).


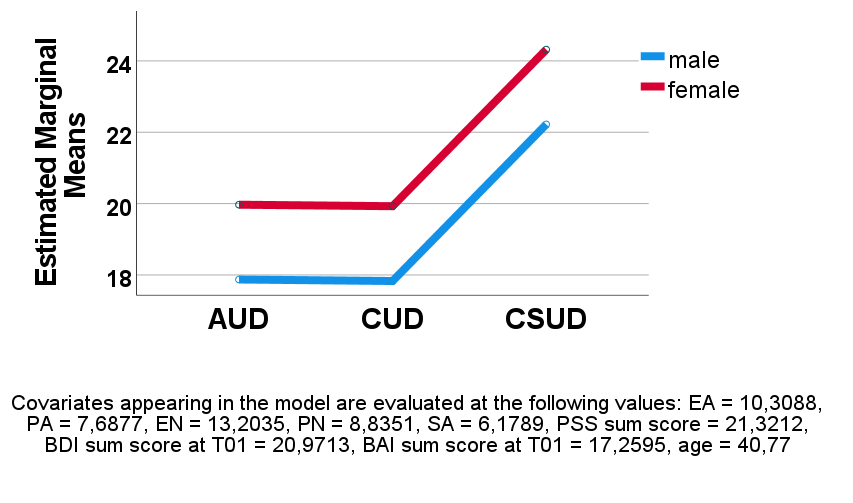


**Supplementary Figure 1: Estimated marginal means of the MACS sum score (craving) at admission to treatment.** After adjusting for all five subscores of CM, depressiveness, anxiety and perceived stress, as well as age, individuals with CSUD reported more severe craving at admission compared to AUD or CUD, to did females compared to males.

**The influence of different types of CM on the reduction of substance craving**

Over all three substance groups, craving diminished from 17.9 (10.0) at T01 to 11.0 (8.4) at T14 in the MACS questionnaire. A significant of sex (F(1, 296) = 4.321, p = 0.039, η² = 0.014) but not substance group (F(2, 296) = 1.331, p = 0.266, η² = 0.009) was observed after adjusting for age and all five subscores of CM. There was no significant influence regarding all subscores of CM. Post-hoc tests regarding sex revealed significantly higher reduction of craving in women compared to men (p = 0.039, M_Diff_ = 2.30, 95%-CI[0.12, 4.47]).

Including PSS, BDI (T01) and BAI (T01), no significant effect of substance group (F(2, 272) = 0.423, p = 0.656, η² = 0.003) or sex (F(1, 272) = 1.228, p = 0.269, η² = 0.004) did emerge. However, PSS, BDI (T01), and BAI (T01) sum scores excerpted a significant influence (F(1, 272) = 13.184, p < 0.001, η² = 0.046; F(1, 272) = 21.677, p < .001, η² = 0.074; F(1, 272) = 3.928, p = 0.048, η² = 0.014), so did age (F(1, 272) = 5.115, p = 0.025, η² = 0.018).

KLINITZKE, G., ROMPPEL, M., HAUSER, W., BRAHLER, E. & GLAESMER, H. 2012. [The German Version of the Childhood Trauma Questionnaire (CTQ): psychometric characteristics in a representative sample of the general population]. *Psychother Psychosom Med Psychol,* 62**,** 47-51.

WINGENFELD, K., SPITZER, C., MENSEBACH, C., GRABE, H. J., HILL, A., GAST, U., SCHLOSSER, N., HOPP, H., BEBLO, T. & DRIESSEN, M. 2010. [The German version of the Childhood Trauma Questionnaire (CTQ): preliminary psychometric properties]. *Psychother Psychosom Med Psychol,* 60**,** 442-50.
